# An integrative, systemic day-clinic approach for the treatment of psychiatric disorders in young adults: a detailed study of two cases

**DOI:** 10.1192/j.eurpsy.2024.1251

**Published:** 2024-08-27

**Authors:** U. Altunoez, A. Osiak, M. Anthoff, G. Rietig, M. Bald, S. Akbas, I. T. Graef-Calliess

**Affiliations:** KRH Wunstorf Psychiatric Hospital, Wunstorf, Germany

## Abstract

**Introduction:**

More than 75% of the psychiatric disorders arise before the age of 30. Adolescence and young adulthood pose numerous developmental challenges like identity development, educational and occupational concerns, gaining autonomy and boundary-setting skills. The adolescent crises, which can involve a broad spectrum of psychiatric symptoms, demands a multidisciplinary approach to diagnosis and treatment.

**Objectives:**

Our goal is to present a best practice example of an interdisciplinary day clinic through two case presentations, aiming improving innovative strategies for assessment/treatment of psychiatric disorders in young adulthood.

**Methods:**

Via two comprehensive case presentations, we will introduce a psychotherapeutic day-clinic concept from a psychiatric training hospital in Germany.

**Results:**

The day clinic’s interdisciplinary team uses therapeutic approaches like dynamic, cognitive-behavioral, and systemic therapy to understand young adults beyond just their symptoms. Milieu-therapeutic methods, family constellations, socio-therapeutic approaches and non-verbal therapies are incorporated into our concept.

Case 1: A 20-year-old male patient, previously diagnosed with schizophrenia, was referred due to symptoms of living in an unreal world with perceived magical abilities and family conflicts. In the evaluation the features of high-functioning autism spectrum disorder (ASD) were more prominent than the psychotic symptoms. Developmental history and diagnostic tools yielded the diagnosis of ASD. Magical abilities in an unreal world appears to align more closely with repetitive/restrictive patterns of behavior, hereby we excluded in the follow-up the diagnosis of schizophrenia. Psychoeducation, social-skills-training and family interventions helped him to comprehend his strengths and discover a clearer direction in his life.

Case 2: Another 20-year-old male patient was referred with depressive symptoms, a sense of emptiness and self-mutilation. Following routine evaluation, we employed systemic methods (genogram constellations) to gain deeper insight into the patient’s psychopathology. His mother’s migration history from Thailand, coupled with unfulfilled aspirations, echoed in his recurring thought: “Where are my roots?” During follow-up, we recognized his passive stance toward therapeutic change, addressed through a systemic intervention known as ‘taking the side of non-change.’ This shifted his position from resistance to openness. Non-verbal approaches, family interventions, and corrective in-vivo experiences significantly contributed to his stabilization.

**Conclusions:**

Specialized psychiatric centers tailored to the unique needs of young adults play a critical role in evaluating, diagnosing, and treating psychiatric crises during this developmental stage. Achieving this requires the implementation of interdisciplinary holistic therapeutic approaches.

**Disclosure of Interest:**

None Declared

